# Identification of Genetic Loci and Candidate Genes Related to Grain Zinc and Iron Concentration Using a Zinc-Enriched Wheat ‘Zinc-Shakti’

**DOI:** 10.3389/fgene.2021.652653

**Published:** 2021-05-31

**Authors:** Nagenahalli Dharmegowda Rathan, Deepmala Sehgal, Karthikeyan Thiyagarajan, Ravi Singh, Anju-Mahendru Singh, Velu Govindan

**Affiliations:** ^1^Indian Agricultural Research Institute, New Delhi, India; ^2^International Maize and Wheat Improvement Center (CIMMYT), Texcoco, Mexico

**Keywords:** wheat, biofortication, zinc, breeding, QTL quantitative trait loci

## Abstract

The development of nutritionally enhanced wheat (*Triticum aestivum* L.) with higher levels of grain iron (Fe) and zinc (Zn) offers a sustainable solution to micronutrient deficiency among resource-poor wheat consumers. One hundred and ninety recombinant inbred lines (RILs) from ‘Kachu’ × ‘Zinc-Shakti’ cross were phenotyped for grain Fe and Zn concentrations and phenological and agronomically important traits at Ciudad Obregon, Mexico in the 2017–2018, 2018–2019, and 2019–2020 growing seasons and Diversity Arrays Technology (DArT) molecular marker data were used to determine genomic regions controlling grain micronutrients and agronomic traits. We identified seven new pleiotropic quantitative trait loci (QTL) for grain Zn and Fe on chromosomes 1B, 1D, 2B, 6A, and 7D. The stable pleiotropic QTL identified have expanded the diversity of QTL that could be used in breeding for wheat biofortification. Nine RILs with the best combination of pleiotropic QTL for Zn and Fe have been identified to be used in future crossing programs and to be screened in elite yield trials before releasing as biofortified varieties. *In silico* analysis revealed several candidate genes underlying QTL, including those belonging to the families of the transporters and kinases known to transport small peptides and minerals (thus assisting mineral uptake) and catalyzing phosphorylation processes, respectively.

## Introduction

About 3 billion people around the world, especially in countries where cereal-based foods represent the largest proportion of the daily diet, suffer from micronutrient malnutrition resulting primarily from iron (Fe) and zinc (Zn) deficiencies (Cakmak, 2002; Bouis and Islam, 2012; Black et al., 2013; Grew, 2018). The Fe and Zn deficiencies affect the immune system and cognitive abilities and are considered to be important causes of retarded growth (Bryan et al., 2004; Stoecker et al., 2009). Approximately one-fourth of the world population suffers from anemia caused by iron deficiency (Allen et al., 2006). The pregnant women and young children are the hardest hit sections to acute micronutrient malnutrition. WHO states that globally 45% of annual child mortality is attributed to malnutrition. In India alone, more than 50% of children below 5 years of age and pregnant women are anemic, whereas 38% of children of the same age group are stunted (Sharma et al., 2020).

Hence, food security is not only about consuming a sufficient quantity of food but also nutrients to ensure proper human health. The process of supplementation and fortification of food products are common practices to reduce micronutrient deficiency. However, supplementation and fortification have not been successful where malnutrition problem is alarming due to the unaffordability of either of the two options. Moreover, these are not sustainable approaches to combat micronutrient malnutrition due to recurring investments (Pfeiffer and McClafferty, 2007). Genetic biofortification, a strategy to develop the staple food crop varieties with increased levels of micronutrients *and* reduced levels of anti-nutrients using plant breeding techniques, has been heralded as a sustainable and long-term solution for contributing to alleviating the malnutrition problem (Bouis and Saltzman, 2017).

Wheat (*Triticum aestivum* L.) is the staple food for over 2.5 billion people, and accounts for 17% of the calories and 20% of human protein intake (Gerard et al., 2020). Wheat is also a main source of micronutrients in predominantly vegetarian populations lacking food diversity in many developing countries (Braun et al., 2010). Therefore, wheat is a suitable candidate for genetic biofortification to combat this hidden hunger among the rural and urban poor particularly from underdeveloped and developing regions of the world.

Genetic variability is the first prerequisite in plant breeding–based methods. *Triticum* species related to wheat such as *Aegilops tauschii*, *Triticum monoccocum*, *Triticum dicoccum*, *Triticum boeticum*, and *Triticum spelta* have been found to be the sources of tremendous genetic diversity for grain Zn and Fe concentrations and for other agronomic and nutritional quality traits (Velu et al., 2014). However, the introgression of genes from these wild relatives to the elite wheat lines requires substantial efforts, time, and resources. Hence, synthetic hexaploids were developed by crossing tetraploid durum wheat (*T. durum* or *T. dicoccum*) with *Ae. tauschii* and synthetic-derived lines have been used extensively in introgressing genes into the elite bread wheat (Ginkel et al., 2002). The introgressions from synthetic hexaploids were exploited to develop varieties like ‘WB02’ and ‘Zinc-Shakti’ with 20–40% increased Zn content than local checks (Velu et al., 2020).

Quantitative trait loci (QTL) mapping using bi- or multi-parental populations is highly useful for the discovery of genes for quantitative traits such as grain Zn and Fe and for the development of molecular markers to be used in breeding programs (Velu et al., 2019). The advances made in next-generation sequencing technologies in wheat and other crops, generating thousands of markers faster and cheaper (Poland et al., 2012; Wang et al., 2014; Winfield et al., 2016), led to increased QTL mapping and genomic prediction studies in the past decade for multiple traits including grain Zn and Fe (Hao et al., 2014; Tiwari et al., 2016; Velu et al., 2017; Liu et al., 2019; Cu et al., 2020). QTL linked to grain Zn and Fe contents have been reported on chromosomes 1A, 2A, 5A, 2B, 3D, 4B, 6A, 6B, and 7A as individual or pleiotropic genomic regions controlling grain Zn and Fe contents and/or thousand kernel weight (Xu et al., 2012; Hao et al., 2014; Crespo-Herrera et al., 2016).

Therefore, identifying the genomic regions that regulate the accumulation of Zn and Fe in the grain without any confounding effects on grain yield would allow breeders to develop high yielding biofortified cultivars. In the current study, we evaluated a recombinant inbred line (RIL) population, developed from a cross between the high grain Zn cultivar Zinc-Shakti and the low Zn cultivar Kachu, for grain Zn and Fe and other agronomic and yield attributing traits. The specific objectives of the study were to (1) identify stable QTL associated with grain iron (GFeC) and zinc (GZnC) concentration for use in wheat breeding programs, (2) identify lines with best QTL combinations to be deployed in future crossing program, and (3) dissect the role of epistasis in the genetic architecture of nutritional traits (GZnC and GFeC).

## Materials and Methods

### Planting Material

The F_6_ population consisting of 190 RILs was developed from the cross between the CIMMYT’s high Zn wheat cultivar Zinc-Shakti and the low Zn cultivar Kachu. The RIL population along with the two parents and two commercial checks, Baj and Borlaug100, were grown at Norman E. Borlaug Research Station, Ciudad Obregon, Sonora, Mexico to evaluate for the agronomic and nutritional quality traits.

### Phenotyping and Analysis of Phenotypic Data

The RIL population was evaluated for grain zinc (GZnC), iron (GFeC) concentration, thousand kernel weight (TKW), and plant height (PH) for 3 consecutive years during 2017–2018 (Y1), 2018–2019 (Y2), and 2019–2020 (Y3). The yield components and agronomic traits were test weight (TW), days to heading (DH), and days to maturity (DM) for 2 consecutive years during 2017–2018 (Y1) and 2018–2019 (Y2). Each RIL was grown on a double-row plot of 1 m length and 0.8 m width in a bed-planting system in randomized complete block design with two replications. Diseases and pests were controlled chemically, whereas weeds were controlled both manually and chemically. Plant materials were harvested after physiological maturity when grains were totally dry in the field. Grain samples of about 20 g for each entry were carefully cleaned to discard broken grains and foreign material and then used for micronutrient analysis. GZnC and GFeC were measured with a “bench-top” non-destructive, energy-dispersive X-ray fluorescence spectrometry (ED-XRF) instrument (model X-Supreme 8000; Oxford Instruments plc, Abingdon, United Kingdom) standardized for high-throughput screening of mineral concentration of whole grain wheat (Paltridge et al., 2012). Two laboratory commercial checks, namely, Baj and Borlaug100, were used as in-house quality control checks. TKW was measured with Seed Count digital imaging system (model SC5000; Next Instruments Pty Ltd., NSW, Australia) that was standardized to measure TKW. The Seed-Count system can rapidly and accurately measure wheat grain samples, determining the grain number and grain physical characteristics based on flatbed scanner technology. The data on DH was measured by counting the number of days from germination to 50% of plants heading in a plot. DM was measured by counting the number of days from germination to physiological maturity when more than 50% of spikes were ripe and had turned yellow. PH was measured from the ground to the tip of the spike excluding awns at the late grain-filling stage.

All phenotypic data analysis was conducted in Meta-R (Multi Environment Trial Analysis with R) version 6.0 software. Best linear unbiased predictors (BLUPs) of each RIL were obtained for an individual year and across years in Meta R. These estimated BLUPs were used in QTL analysis. The broad sense heritability (*h*^2^) was also estimated in Meta R for traits in each year and across years.

### Genotyping

The genomic DNA was extracted by following the standard procedures by Dreisigacker et al. (2004). The population was genotyped with the DArTSeq technique (Edet et al., 2018) in the Genetic Analysis Service for Agriculture (SAGA) with current headquarters at CIMMYT, El Batan, Texcoco, Estado de Mexico. The array technology works on the genome complexity reduction concept by using a combination of restriction enzymes to obtain a representation of the whole genome. The FASTQ files were quality filtered using a Phred quality score of 30. More stringent filtering was also performed on barcode sequences using a Phred quality score of 10, which represents 99.9% of base call accuracy for at least 75% of the bases. A proprietary analytical pipeline developed by DArT P/L was used to generate allele calls for SNP and presence/absence variation (PAV) markers.

### Linkage Mapping, QTL Analysis, and Epistatic Interactions

The initial genotypic information consisted of 40,059 markers codified as 0 and 1 for the two homozygous parental alleles and 2 for the heterozygotes. Parental non-polymorphic markers, markers with >30% missing data, and the redundant markers were discarded using bin function in QTL IciMappingv4.2 software. A filtered set of 909 highly informative DArTseq markers were used for linkage map construction. The linkage groups were assembled from the genotypic data using QTL IciMappingv4.2 software^1^, applying a LOD threshold of 3.0 between adjacent markers (Liu et al., 2019). Markers were ordered with the nnTwoOpt algorithm, using a 5 cM window size for rippling the markers in each linkage group. Linkage groups with less than five markers were discarded from the analysis. QTL were identified using two approaches: inclusive composite interval mapping (ICIM) and multi-environment QTL mapping, both algorithms implemented in QTL IciMappingv4.2 software. ICIM identifies additive and dominant QTL for single environment. The multi-environment QTL mapping uses a QTL by environment interaction (QEI) model and estimates both additive and additive × year interaction effects for each QTL. This methodology first conducts stepwise regression in each environment to identify the most significant marker variables and then one-dimensional scanning on the adjusted phenotypic values across the environments to detect QTL with both average effect and QEI effects. A LOD threshold of 2.5 was applied to call significance. QTL nomenclature was done following the standard procedure^2^.

We estimated two- and three-locus epistatic interactions among identified QTL in R using a script described in Sehgal et al. (2017). Marker–marker interactions were declared significant at a threshold of *p* < 0.0001 and R^2^ was used to describe percentage variation explained (PVE) by the significant interactions.

### *In silico* Analysis of QTL

After the identification of QTL, an *in silico* search of the candidate genes was conducted in the Ensemble Plants database^3^ of the bread wheat genome with the Basic Local Alignment Search Tool (BLAST) using the sequence information of the markers present within the peak of the QTL and the flanking markers.

### QTL Additive Effects Estimation

The two genotypic classes of the flanking markers of all important QTL (with highest PVE and pleiotropic for GZnC and GFeC) were compared for the average trait value. Additive effects were then estimated for multiple QTL combinations. The RILs with the best combination of QTL for GZnC and GFeC were identified by estimating the additive effects of multiple QTL.

## Results

### Phenotypic Evaluations

**FIGURE 1:**
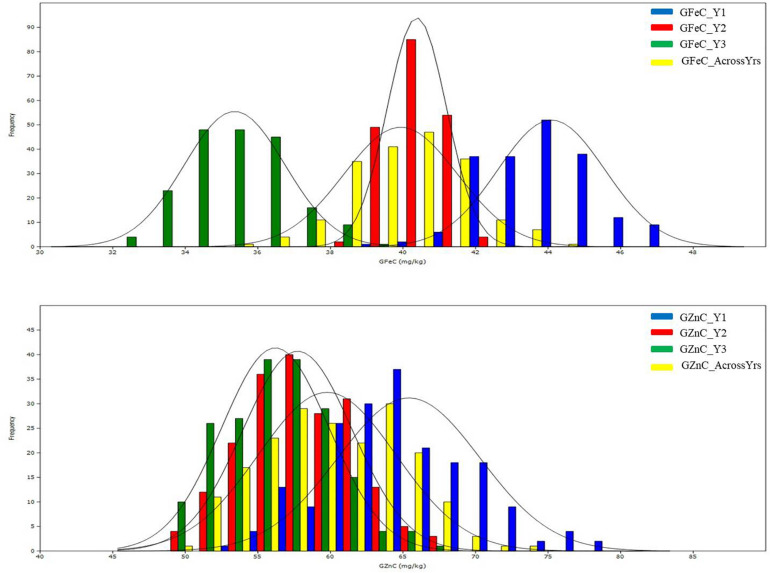
Histograms of grain zinc (GZnC) and iron (GFeC) concentration in the mapping populations of recombinant inbreed lines evaluated during 3 years and across the years.

The mean values of the traits for the two parents, Kachu and Zinc-Shakti, RILs, and the two checks, namely, Baj and Borlaug100, in year I (Y1), year II (Y2), year III (Y3), and across years are shown in Table 1. Large and significant differences were observed between the parents for all the traits except TKW. The RIL population showed a normal to near normal frequency distribution for all the traits in all the years and across the years (Figure 1 and Supplementary Figures 1–5). The range, mean, coefficient of variation (CV), heritability, and variance estimates for the RIL population provided in Table 1. Transgressive segregants for GZnC and GFeC were obtained in the RIL population. For GZnC, the highest performing three RILs showed average values of 70.8, 69.4, and 68.4 mg/kg, which are 9.4 to 15.6 mg/kg higher than the two parents and checks. For GFeC, the three RILs showed average values of 42.9, 42.7, and 42.5 mg/kg, which are 2.9 to 5.0 mg/kg higher than the two parents and checks.

**TABLE 1:** Phenotypic values, mean, range, CV, heritability, and variance estimates of the traits in RILs, parents, and two commercial checks evaluated during 2017–2018 (Y1), 2018–2019 (Y2), 2019–2020 (Y3), and across the years at Ciudad Obregon, Mexico.

Trait	Environment	Kachu (P1)	Zinc-Shakti (P2)	RIL population						Baj (C1)	Borlaug100 (C2)
				Range	Mean	CV (%)	LSD	H2 (BS)	Genotypic variance		
GZnC (mg/kg)	Y1**	55.6	65.4	53.1–80.2	65.4	7.47	8.25	0.73	32.89***	57.2	60.8
	Y2**	44.5	58	50.1–67.5	57.7	8.71	7.92	0.64	22.27***	52.9	56.9
	Y3**	49.9	57.5	48.5–68.1	56.2	7.53	6.97	0.69	19.98***	52.9	55.6
	Across years*	50	60.3	50.4–72.9	59.8	7.8	4.99	0.88	25.67***	52.9	57.3
GFeC (mg/kg)	Y1*	40.5	46.4	39.5–47.8	44.1	6.48	4.03	0.51	4.28***	41.8	42.5
	Y2*	37.5	42.1	38.6–42.7	40.4	6.89	3.2	0.34	1.99**	38.9	40.1
	Y3*	31.5	34.5	31.9–39.1	35.4	5.7	3.12	0.61	3.16***	33.2	34.1
	Across years*	36.5	41	35.3–43.9	39.9	6.4	2.52	0.75	3.27***	36.8	38.5
TKW (g)	Y1	48.4	47.5	39.6–59.1	48.3	2.32	2.15	0.95	11.28***	52.7	53.6
	Y2	44.7	45.5	40.3–58.1	47.6	4.02	3.44	0.83	8.93***	50.6	51.1
	Y3	44.6	43.6	35.5–54.2	46.1	3.4	2.87	0.86	7.65***	48.8	51.5
	Across years	45.9	45.5	38.9–57.9	47.3	3.31	1.91	0.95	9.02***	50.9	52.4
TW (g)	Y1*	79.8	76.2	75.7–81.5	78.6	0.76	1.06	0.81	0.78***	79.8	79.7
	Y2*	78.2	74.8	74.8–78.0	77.1	3.31	2.17	0.19	0.75	77.5	77.4
	Across years*	79	75.5	75.0–79.7	77.9	2.38	1.77	0.46	0.75***	78.7	78.6
DH (days)	Y1	81	74	67.1–98.5	80.2	3.24	4.92	0.92	40.84***	72.2	80
	Y2**	84.5	70.5	69.6–103.5	84.1	3.41	5.44	0.93	53.22***	76.1	81.7
	Across years*	82.8	72.3	68.1–101.1	82.1	3.32	3.95	0.96	46.58***	73.9	80.8
DM (days)	Y1*	127.5	119	113.4–136.9	124.4	2.05	4.74	0.89	26.11***	114.3	125.4
	Y2	134	131	120.9–138.3	129.9	2.18	5.08	0.83	19.30***	120.9	128.7
	Across years	130.8	125	117.0–137.9	127.1	2.12	4.02	0.91	21.81***	117	127
PH (cm)	Y1*	82.8	89.5	80.9–113.0	94.2	3.52	6.21	0.9	51.15***	87.2	85.9
	Y2*	97	103	90.1–117.9	105.7	0.66	1.39	0.99	36.57***	101	100
	Y3**	97.5	107.5	89.7–115.4	101.3	1.14	2.24	0.98	31.32***	93.2	92.2
	Across years**	92.4	100.3	89.7–112.7	100.4	2.06	5.76	0.84	26.48***	94.6	93.6

The *t*-test results indicating significant difference between parents for each trait are provided in the environment column. *Significant values at *p* < 0.05, **significant values at *p* < 0.01, ***significant values at *p* < 0.001.

**FIGURE 2:**
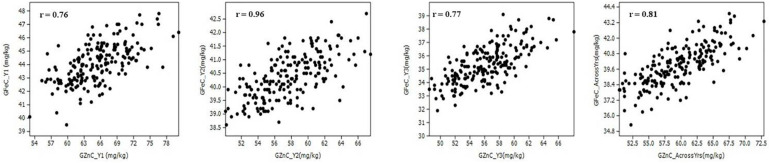
Scatter plots showing the correlation of grain zinc (GZnC) and iron (GFeC) concentration among recombinant inbreed lines evaluated during 3 years and across the years.

The Pearson correlation coefficients (*r*) observed in the population between GZnC and GFeC were statistically significant (*p* < 0.001) in each year of evaluation and across years (Figure 2 and Table 2). GZnC is found to have a consistently significant negative correlation with DH and DM at *p* < 0.05 to 0.001, whereas it exhibited a significant positive correlation with TKW (*p* < 0.001) in Y2, Y3, and across years. GFeC showed a significant positive correlation with PH in Y1, Y2, and across years (*p* < 0.05 to 0.001) and a negative association with TW in Y2 and over the years (*p* < 0.001). The DH, DM, and PH exhibited a highly significant correlation with each other in each year and also across the years (*p* < 0.001). TKW showed a consistently negative correlation with DH, DM, and PH in all the environments (Table 2).

**TABLE 2:** Pairwise phenotypic correlation coefficients among traits in RIL population in 2017–2018 (Y1), 2018–2019 (Y2), 2019–2020 (Y3), and across the years.

	Traits	DH	DM	PH	TKW	TW	GZnC
	DM	0.93***					
	PH	0.61***	0.44***				
2017–2018 (Y1)	TKW	−0.48***	−0.55***	−0.31***			
	TW	–0.14	–0.12	0.13	0.04		
	GZnC	–0.14	−0.18*	–0.02	0.11	0.06	
	GFeC	0.25***	0.24***	0.40***	−0.17*	0.06	0.76***
	DM	0.90***					
	PH	0.49***	0.41***				
2018–2019 (Y2)	TKW	−0.42***	−0.55***	−0.20**			
	TW	−0.28***	−0.36***	–0.12	−0.20**		
	GZnC	−0.30***	−0.42***	–0.03	0.39***	−0.39***	
	GFeC	–0.03	−0.23**	0.15*	0.24***	−0.91***	0.96***
	DM	–					
	PH	–	–				
2019–2020 (Y3)	TKW	–	–	–0.09			
	TW	–	–	–	–		
	GZnC	–	–	–0.01	0.32***	–	
	GFeC	–	–	0.12	0.28***	–	0.77***
	DM	0.94***					
	PH	0.77***	0.63***				
Across years	TKW	−0.44***	−0.54***	−0.27***			
	TW	−0.18*	−0.19**	0.21**	–0.03		
	GZnC	−0.18*	−0.26***	–0.01	0.27***	–0.07	
	GFeC	0.09	0.01	0.20**	0.10	−0.29***	0.81***

*Significant at *p* < 0.05, **significant at *p* < 0.01, ***significant at *p* < 0.001.

### Linkage Map Construction and QTL Analysis

The linkage map representing all the wheat chromosomes was constructed using 909 non-redundant filtered markers. The number of markers in the linkage groups ranged from 5 (5D) to 109 (2B), whereas the map distance of the linkage groups ranged between 24 cM (1D) and 537 cM (2A). Among the total number of markers, 45.32 and 40.70% of markers were grouped on the A and B genome, respectively, whereas the D genome had the least number of markers (13.97%; Table 3). The whole linkage map covered a genetic distance of 4665 cM with an average inter-marker distance of 5.13 cM.

**TABLE 3:** Percentage of markers grouped by each wheat chromosome and genome in the RIL mapping population.

Genome	RIL population							
Chromosome	1	2	3	4	5	6	7	Total
**A**	6.82	8.80	5.94	7.37	4.95	7.37	4.07	45.32
**B**	5.06	11.99	5.06	0.99	9.90	3.41	4.29	40.70
**D**	1.21	1.87	4.18	0.11	0.55	1.32	4.73	13.97

Considering all environments individually, QTL mapping using ICIM identified a total of 15 QTL each for GZnC and GFeC. Of these, nine and four QTL for GZnC and GFeC, respectively, were detected in at least two environments (Table 4) and thus were stable. The nine stable QTL detected for GZnC showed considerable variation in LOD scores and percentage variation explained (PVE) in different environments. For example, QTL detected on chromosomes 2A and 2B showed the highest LOD (11.0 and 9.2) and PVE (10.3 and 13.3%) in one of the environments and a moderate value for both LOD and PVE in the remaining environments. The QTL on chromosome 7D, on the other hand, showed moderate values for both LOD scores (6.7–8.0) and PVE (7.9–9.5%) in all environments where it was detected. The QTL on chromosome 1D was detected in all four environments; however, it had minor effects in one of the environments (PVE of 5.4% in environment 2). Out of four stable QTL detected for GFeC, the one on chromosome 2A was identified in all four environments with moderate to high LOD scores (5.2–9.4) but consistently high PVE (>10.0%) in all environments.

**TABLE 4:** Summary of the stable QTL identified from the ICIM analysis detected at least in two environments for GZnC and GFeC.

QTL name	Envi	Chr	Flanking markers	LOD	PVE (%)	Add	CI
**Grain zinc concentration (GZnC)**							
QZnC-1D	1,2,3,4	1D	1318890–1167672	5.2, 6.2,2.8, 9.1	7.1, 5.4, 7.6, 8.6	−1.34, −0.96, −1.13, −1.48	0–43
QZnC-1B.1	1,2,4	1B	1132017–4909722	5.6, 7.0, 8.4	7.8, 6.3, 7.5	−1.39,−1.04,−1.38	79.5–82.5
QZnC-1B.2	1,2,4	1B	13142877–3954275	3.4, 5.9, 8.2	6.2, 5.3, 9.1	1.25, 0.95, 1.53	317.5–326.5
QZnC-2B	2,3,4	2B	993617–993562	9.2, 8.2, 3.8	8.7, 13.3, 3.2	−1.22,−1.51,−0.92	251.5–255.5
QZnC-7D.1	1,2,4	7D	100024878–5050443	6.7, 6.8, 8.0	9.5, 8.9, 7.9	1.56, 1.25, 1.43	105.5–111.5
QZnC-7D.2	1,4	7D	4910838–1211477	2.6, 3.9	3.6, 3.3	−0.97,−0.94	164.5–167.5
QZnC-2A	2,4	2A	1111617–982253	11.0, 7.6	10.3, 6.8	−1.32,−1.33	129.5–133.5
QZnC-5A	2,4	5A	994618–1135154	6.0, 8.4	5.5, 8.2	−0.99,−1.47	126.5–127.5
QZnC-6B	2,4	6B	2280881–3024410	4.0, 3.9	3.6, 3.6	−0.79, −0.96	210.5–215.5
**Grain iron concentration (GFeC)**							
QFeC-2A	1,2,3,4	2A	1195992–2300803	9.4, 5.2, 6.7, 8.3	11.4, 10.2, 10.1, 10.6	−0.56,−0.26,−0.45,−0.53	104.5–148.5
QFeC-1D	1,3,4	1D	1318890–1167672	2.8, 8.0, 5.1	3.1, 12.3, 6.1	−0.3, −0.5, −0.4	0–43
QFeC-1B	2,3	1B	1240883–1005607	2.8, 4.1	5.9, 5.9	0.2, 0.34	34.5–43.5
QFeC-6A	1,4	6A	1698406–100027274	5.4, 8.6	6.3, 10.9	−0.41, −0.54	110.5–112.5

*1—Y1, 2—Y2, 3—Y3, 4—Across years.*

**FIGURE 3:**
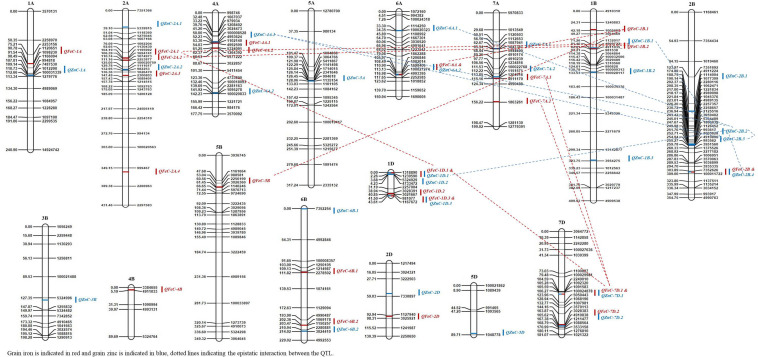
The QTLs for grain zinc (GZnC) and iron (GFeC) concentration on genetic map from the RIL population derived from Kachu and Zinc-Shakti.

Quantitative trait loci analysis using multi-environment QTL model detected 27 QTL for GZnC on chromosomes 1A, 2A, 4A, 5A, 6A, 7A, 1B, 2B, 3B, 6B, 1D, 2D, 5D, and 7D with PVE ranging from 1.1 to 8.1%. Chromosomes 1B, 1D, and 2B localized multiple (more than two) QTL for GZnC (Figure 3). The favorable alleles at 16 QTL regions were contributed by the parent Zinc-Shakti, whereas at 11 QTL regions these were contributed by Kachu. Table 5 shows details of all QTL along with LOD scores for the additive average effect and for G × Y interaction. Two QTL for GZnC, *QZnC-1B.1* and *QZnC-7D.1*, explained the highest PVE of 7.7 and 8.1%, respectively. Four QTL, *QZnC-2A.2*, *QZnC-1B.3*, *QZnC-2B.3*, and *QZnC-1D.2*, showed moderate PVE (>5% but <7.0%). For GFeC, multi-environment model revealed 23 QTL on chromosomes 1A, 2A, 4A, 6A, 7A, 1B, 2B, 4B, 5B, 6B, 1D, 2D, and 7D with PVE ranging from 1.0 to 10.2%. The favorable alleles at 14 QTL regions were contributed by the parent Zinc-Shakti, whereas at 9 QTL regions these were contributed by Kachu (Table 5). Three QTL, *QFeC-2A.2*, *QFeC-6B.1*, and *QFeC-1D.3*, explained the highest PVE of 10.1, 10.2, and 7.3%, respectively. Three QTL, *QFeC-2A.1*, *QFeC-6A*, and *QFeC-5B*, explained moderate PVE of 5.9, 5.6, and 5.9%, respectively. It is noteworthy that all stable QTL detected for GZnC and GFeC by ICIM analysis were also detected in multi-environment analysis.

**TABLE 5:** Summary of the QTL identified from the multi-environment QTL analysis for GZnC and GFeC.

QTL	Chr	Position	Flanking markers	LOD	LOD (G × E)	PVE	PVE (G × E)	Add	Add × Y1	Add × Y2	Add × Y3	CI
**Grain zinc concentration (GZnC)**												
QZnC-1A	1A	111	1012290–100031339	4.94	0.99	3.7	1.51	–0.57	–0.64	0.46	0.18	110.5–112.5
QZnC-2A.1	2A	39	7351366–5339915	2.7	1.03	1.6	0.72	0.36	0.4	–0.01	–0.39	29.5–42.5
QZnC-2A.2*	2A	132	1111617–982253	13.89	4.44	6.1	0.78	–0.86	0.14	–0.45	0.31	129.5–133.5
QZnC-4A.1	4A	52	100008528–4993624	4.15	0.8	2.4	0.5	0.51	–0.16	–0.21	0.37	50.5–52.5
QZnC-4A.2	4A	142	1095278–100020833	4.11	0.7	2.2	0.31	0.51	0.25	0.01	–0.26	140.5–144.5
QZnC-5A*	5A	127	994618–1135154	6.57	3.31	2.6	0.69	–0.52	0.14	–0.44	0.3	126.5–127.5
QZnC-6A.1	6A	34	1114250–100020323	2.51	2.36	1.3	1.25	0.11	–0.59	0.24	0.35	22.5–37.5
QZnC-6A.2	6A	111	1698406–100027274	3.56	0.35	1.8	0.05	–0.5	0.05	0.07	–0.12	110.5–112.5
QZnC-7A.1	7A	63	1087941–1047387	4.92	0.46	3.6	1.06	0.59	0.52	–0.39	–0.13	62.5–63.5
QZnC-7A.2	7A	112	100022768–1151571	2.76	0.08	1.5	0.06	–0.45	–0.13	0.09	0.03	111.5–113.5
QZnC-1B.1*	1B	81	1132017–4909722	13.67	2.17	7.7	0.98	–0.96	–0.41	–0.07	0.48	79.5–82.5
QZnC-1B.2	1B	131	100009313–100028117	2.62	0.88	1.1	0.1	–0.37	0.16	–0.14	–0.02	126.5–133.5
QZnC-1B.3*	1B	323	13142877–3954275	9.83	2.16	5.1	0.65	0.79	0.27	0.15	–0.42	317.5–326.5
QZnC-2B.1	2B	189	1114004–1077309	6.75	4.04	2.6	1.13	0.46	–0.52	0.47	0.05	187.5–190.5
QZnC-2B.2*	2B	252	993617–1003628	10.1	5.64	4	1.53	–0.59	0.09	–0.61	0.51	251.5–252.5
QZnC-2B.3*	2B	255	4910896–993562	8.84	3.48	5.7	2.73	–0.64	0.19	0.64	–0.83	254.5–255.5
QZnC-2B.4	2B	305	7352626–100011722	5.58	1.52	3.2	0.98	0.57	–0.26	–0.27	0.53	301.5–308.5
QZnC-3B	3B	129	5324996–1255832	2.5	0.38	1.3	0.13	–0.4	0.1	0.09	–0.19	116.5–137.5
QZnC-6B.1	6B	0	7352254–4992846	2.51	0.53	1.4	0.26	–0.39	0.14	0.13	–0.27	0–17.5
QZnC-6B.2*	6B	212	2280881–3024410	5.97	1.61	2.8	0.37	–0.58	–0.15	–0.17	0.32	210.5–215.5
QZnC-1D.1*	1D	0	1318890–1239590	8.03	3.67	3.3	0.82	–0.58	0.44	–0.38	–0.06	0–1.5
QZnC-1D.2*	1D	4	5324929–4733472	7.5	1.63	5.5	2.19	–0.68	–0.65	0.7	–0.05	3.5–4.5
QZnC-1D.3*	1D	43	981077–1167672	4.08	0.16	2.2	0.09	–0.54	–0.03	0.16	–0.12	41.5–43
QZnC-2D	2D	53	3222503–7330897	2.61	0.33	1.3	0.13	0.4	0.17	–0.02	–0.15	30.5–58.5
QZnC-5D	5D	88	1093565–1048778	2.8	1.25	1.7	0.84	0.34	0.44	–0.04	–0.4	46.5–89
QZnC-7D.1*	7D	107	100024878–5050443	13.59	2.33	8.1	1.48	0.96	0.56	0	–0.56	105.5–111.5
QZnC-7D.2*	7D	166	4910838–1211477	5.67	0.18	3.3	0.27	–0.66	–0.28	0.16	0.12	164.5–167.5
**Grain iron concentration (GFeC)**												
QFeC-1A	1A	91	1120651–1098230	5.36	2.65	2.4	0.5	–0.14	0.06	–0.1	0.04	89.5–91.5
QFeC-2A.1*	2A	106	1195992–1694741	7.76	3.34	5.9	2.88	–0.18	0.16	0.08	–0.24	104.5–107.5
QFeC-2A.2*	2A	111	1074973–2253877	10.2	3.68	10.1	5.59	–0.22	–0.34	0.14	0.2	110.5–111.5
QFeC-2A.3*	2A	145	1026470–2300803	5.53	2.47	2.6	0.39	–0.15	0.02	–0.09	0.07	137.5–148.5
QFeC-2A.4	2A	353	999467–2266963	2.92	0.19	1.9	0.25	0.13	0.01	–0.06	0.06	335.5–384.5
QFeC-4A.1	4A	54	1102140–4992599	3.45	0.26	2.3	0.12	0.15	0.05	–0.02	–0.03	52.5–55.5
QFeC-4A.2	4A	62	5324091–1264392	4.19	0.28	2.7	0.06	0.17	0.03	–0.03	–0.01	60.5–64.5
QFeC-6A*	6A	111	1698406–100027274	6.12	1.42	5.6	2.39	–0.18	–0.22	0.11	0.11	110.5–112.5
QFeC-7A.1	7A	115	1204916–4911170	5.2	0.59	4.6	1.47	–0.18	–0.17	0.12	0.05	113.5–115.5
QFeC-7A.2	7A	156	4990488–1863261	5.62	2.52	4.3	2.2	–0.15	0.12	0.09	–0.21	149.5–161.5
QFeC-1B.1*	1B	41	1240883–1005607	7.03	2.59	4.1	1.1	0.18	–0.14	0.02	0.12	34.5–43.5
QFeC-1B.2	1B	82	4909722–4911035	6.91	1.04	3.9	0.01	–0.2	0	–0.01	0.01	81.5–83.5
QFeC-2B	2B	304	7352626–100011722	2.98	1.23	1.5	0.29	0.11	–0.08	0.04	0.04	300.5–313.5
QFeC-4B	4B	4	3384655–4911033	2.55	0.7	1.4	0.16	0.11	–0.05	0.01	0.05	0–14.5
QFeC-5B	5B	61	2256199–3026360	6.22	2.16	5.9	3.14	0.17	0.26	–0.12	–0.14	59.5–61.5
QFeC-6B.1	6B	110	1214987–2278502	10.43	3.46	10.2	5.2	–0.23	–0.33	0.14	0.19	108.5–111.5
QFeC-6B.2	6B	203	1069178–1150257	4.07	2.2	2.6	1.41	–0.11	0.15	0.01	–0.15	200.5–203.5
QFeC-1D.1*	1D	0	1318890–1239590	3.49	0.07	2.6	0.34	–0.15	–0.04	0.08	–0.05	0–3.5
QFeC-1D.2*	1D	40	3028391–3021667	3.08	0.93	2.6	1.22	–0.12	–0.16	0.07	0.09	35.5–40.5
QFeC-1D.3*	1D	43	981077–1167672	9.52	4.68	7.3	4.17	–0.18	0.19	0.11	–0.29	41.5–43
QFeC-2D	2D	94	1127940–3025921	4.42	0.67	3.4	0.92	0.16	0	–0.12	0.12	91.5–98.5
QFeC-7D.1	7D	108	100024878–5050443	2.53	1.36	1	0.27	0.09	–0.07	0.06	0.01	105.5–120.5
QFeC-7D.2	7D	165	3028383–4910838	4.44	1.44	3.2	1.11	–0.15	0.1	0.05	–0.15	163.5–167.5

*Denotes QTL detected both in ICIM analysis and multi-environment QTL analysis.

Seven pleiotropic QTL intervals were identified for GZnC and GFeC on chromosomes 1B, 1D, 2B, 6A, and 7D (Figure 3). The pleiotropic QTL on chromosome 1B for GZnC (*QZnC-1B.1*) and GFeC (*QFeC-1B.2*) was identified between the interval 79.5 and 83.5 cM and explained 7.7 and 3.9% PVE for GZnC and GFeC, respectively. The favorable allele was contributed by the parent Zinc-Shakti for both GZnC and GFeC at this QTL. Two pleiotropic QTL were detected on chromosome 1D; the first (*QZnC-1D.1* and *QFeC-1D.1*) in the interval from 0 to 3.5 cM explained 3.3 and 2.6% of PVE for GZnC and GFeC, respectively, and second (*QZnC-1D.3* and *QFeC-1D.3*) in the interval from 41.5 to 43.0 cM explained 2.2 and 7.3% of PVE, respectively. On chromosome 2B, the fourth pleiotropic QTL (*QZnC-2B.4* and *QFeC-2B*) between interval 300.5 and 313.5 cM had minor effects on both GZnC and GFeC and favorable allele was contributed by Kachu for both traits. The pleiotropic QTL on chromosome 6A (*QZnC-6A.2* and *QFeC-6A*) between the interval 110.5 and 112.5 cM had minor (PVE of 1.8%) to moderate (PVE of 5.6%) effects on GZnC and GFeC, respectively. On chromosome 7D, two pleiotropic QTL were localized for GZnC and GFeC; the first detected between the interval 105.5 and 120.5 cM had major effect on GZnC (PVE of 8.1%) and minor effect on GFeC (PVE of 1.0%) whereas the second QTL between the interval 163.5 and 167.5 cM had moderate effects on both traits (PVE of 3.3 and 3.2%, respectively).

Supplementary Table 1 lists the QTL detected for agronomic traits. Briefly, 26, 9, 11, 8, and 23 QTL were detected for TGW, TW, DH, DM, and PH, respectively. Six pleiotropic QTL were detected for nutritional traits and agronomic traits on chromosomes 5A, 6A, 6B, 7A, and 7D. Two such pleiotropic QTL were detected for GZnC, GFeC, and TKW on chromosomes 6A (*QZnC-6A.2*, *QFeC-6A*, and *QTKW-6A.3*) and 7D (*QZnC-7D.1*, *QFeC-7D.1*, and *QTKW-7D.1*) between the intervals 110.5–112.5 and 105.5–120.5 cM, respectively.

### RILs With Best QTL Combinations for Grain Zn and Fe Concentration

The additive effects of the QTL with the highest PVE and of pleiotropic QTL were investigated for GZnC and GFeC (Table 6). The three-QTL combination, viz., *QZnC-2A.2* + *QZnC-1B.1* + *QZnC-7D.1*, showed the highest average GZnC across environments and three RILs were identified with this combination. The two-QTL combination, viz., *QFeC-2A.2* + *QFeC-1D.3*, showed the highest average GFeC across environments and this combination was identified in 15 RILs. The three-pleiotropic-QTL combination (*QZnC-6A.2* + *QFeC-6A _+_ QZnC-1D.3* + *QFeC-1D.3* + *QZnC-7D.1* + *QFeC-7D.1*) showed the highest average GZnC and GFeC across environments and nine RILs were identified with this combination.

**TABLE 6:** The RILs with best QTL combinations for GZnC and GFeC.

QTL	Markers	Marker type	No. of RILs	GZnC (mg/kg)			GFeC (mg/kg)		
				Y1	Y2	Y3	Y1	Y2	Y3
**QTL additive effect for grain zinc concentration (GZnC)**									
QZnC-2A.2	1111617 + 982253	B + B	78	66.43	58.76	56.96	–	–	–
QZnC-1B.1	1132017 + 4909722	B + B	56	67.23	58.63	56.53	–	–	–
QZnC-7D.1	100024878 + 5050443	A + A	39	66.96	59.76	57.20	–	–	–
QZnC-2A.2 + QZnC-1B.1	1111617 + 982253 + 1132017 + 4909722	B + B + B + B	21	68.34	59.49	56.96	–	–	–
QZnC-2A.2 + QZnC-7D.1	1111617 + 982253 + 100024878 + 5050443	B + B + A + A	15	69.84	62.69	59.53	–	–	–
QZnC-1B.1 + QZnC-7D.1	1132017 + 4909722 + 100024878 + 5050443	B + B + A + A	8	69.77	60.67	57.69	–	–	–
QZnC-2A.2 + QZnC-1B.1 + QZnC-7D.1**	1111617 + 982253 + 1132017 + 4909722 + 100024878 + 5050443	B + B + B + B + A + A	3	71.31	63.56	59.21	–	–	–
**QTL additive effect for grain iron concentration (GFeC)**									
QFeC-2A.2	1074973 + 2253877	B + B	50	–	–	–	44.43	40.60	57.38
QFeC-6B.1	1214987 + 2278502	A + A	61	–	–	–	43.59	40.29	56.42
QFeC-1D.3	981077 + 1167672	B + B	51	–	–	–	44.55	40.59	57.48
QFeC-2A.2 + QFeC-6B.1	1074973 + 2253877 + 1214987 + 2278502	B + B + A + A	16	–	–	–	43.94	40.35	56.50
QFeC-2A.2 + QFeC-1D.3**	1074973 + 2253877 + 981077 + 1167672	B + B + B + B	15	–	–	–	44.35	40.63	58.39
QFeC-6B.1 + QFeC-1D.3	1214987 + 2278502 + 981077 + 1167672	A + A + B + B	21	–	–	–	44.42	40.52	57.82
QFeC-2A.2 + QFeC-6B.1 + QFeC-1D.3	1074973 + 2253877 + 1214987 + 2278502 + 981077 + 1167672	B + B + A + A + B + B	9	–	–	–	44.32	40.37	57.09
**Pleiotropic QTL for grain Zn and Fe concentration**									
QZnC-6A.2 & QFeC-6A	1698406 + 100027274	B + B	72	67.42	59.03	57.41	44.43	40.64	57.41
QZnC-1D.3 & QFeC-1D.3	981077 + 1167672	B + B	51	67.00	58.80	57.48	44.55	40.59	57.48
QZnC-7D.1 & QFeC-7D.1	100024878 + 5050443	A + A	39	66.96	59.76	57.20	44.04	40.77	57.20
(QZnC-6A.2 & QFeC-6A) + (QZnC-1D.3 & QFeC-1D.3)	1698406 + 100027274 + 981077 + 1167672	B + B + B + B	33	68.25	59.38	58.14	44.82	40.83	58.14
(QZnC-6A.2 & QFeC-6A) + (QZnC-7D.1 & QFeC-7D.1)	1698406 + 100027274 + 100024878 + 5050443	B + B + A + A	17	68.08	60.33	57.86	44.37	41.03	57.86
(QZnC-1D.3 & QFeC-1D.3) + (QZnC-7D.1 & QFeC-7D.1)	981077 + 1167672 + 100024878 + 5050443	B + B + A + A	15	66.52	60.73	57.60	44.11	40.74	57.60
(QZnC-6A.2 & QFeC-6A) + (QZnC-1D.3 & QFeC-1D.3) + (QZnC-7D.1 & QFeC-7D.1)**	1698406 + 100027274 + 981077 + 1167672 + 100024878 + 5050443	B + B + B + B + A + A	9	69.07	62.37	59.03	44.70	41.34	59.03

**Best combination of QTL, A—Parent 1 (Kachu) type, B—Parent 2 (Zinc-Shakti) type.

### Epistatic Interaction Analysis

For both nutritional traits, epistatically interacting QTL were obtained; two QTL interactions were more frequent than three QTL interactions (Figure 3 and Supplementary Table 2). For GZnC, multiple QTL identified on chromosome 2B interacted significantly with each other, and with QTL identified on chromosomes 1B, 4A, and 6A with PVE ranging from 2.1 to 3.2% (Supplementary Table 2). Besides, significant three-QTL interactions were also observed among QZnC-1D.1, QZnC-2B.2, QZnC-6A.1 and QZnC-2B.4, QZnC-2B.2, QZnC-6A.1 with PVE of up to 4.6%. For GFeC, four epistatically interacting QTL were identified in two-QTL interactions and two in three-QTL interactions. However, PVE by them remained low, from 1.3 to 1.9%.

### *In silico* Analysis

*In silico* analysis identified 13 candidate genes underlying six QTL with highest PVE for GZnC and GFeC (Table 7). The most significant of these are those coding for ABC transporters (*TraesCS2A02G110200* underlying QTL *QZnC-2A.2*) and oligopeptide transporters (*TraesCS7D02G099500* and *TraesCS7D02G139600* underlying *QZnC-7D.1*) playing critical roles in the transport of small peptides, secondary amino acids, and mineral uptake or the kinase-like superfamily, catalyzing phosphorylation processes (*TraesCS1B02G357900* underlying *QZnC-1B.1*).

**TABLE 7:** Putative candidate genes for grain zinc (GZnC) and iron (GFeC) concentration found in the RIL population.

QTL	Chr	Physical distance (Mb)	TraesID	Putative candidate genes	Mol function
QZnC-2A.2	2A	58–62.2	TraesCS2A02G110200	ABC transporter-like, P-loop containing nucleoside triphosphate hydrolase	ATPase-coupled transmembrane transporter activity
			TraesCS2A02G105500	Thioredoxin-like superfamily, EGF-like calcium-binding, conserved site, PA domain	Calcium ion binding
			TraesCS2A02G105600	B3 DNA binding domain, zinc finger, CW-type	DNA binding, zinc ion binding
QZnC-1B.1	1B	531.7–587.7	TraesCS1B02G357900	Serine-threonine/tyrosine-protein kinase, catalytic domain, malectin-like domain, leucine-rich repeat	Protein kinase activity, protein binding, ATP binding
			TraesCS1B02G309100	Protein of unknown function DUF688	–
QZnC-7D.1	7D	59.5–89.4	TraesCS7D02G099500	Oligopeptide transporter, OPT superfamily	Transmembrane transport
			TraesCS7D02G139600	Proton-dependent oligopeptide transporter family, MFS transporter superfamily	Transmembrane transporter activity
QFeC-2A.2	2A	98.2–101.5	TraesCS2A02G150500	Pentatricopeptide repeat, tetratricopeptide-like helical domain superfamily, DYW domain	Protein binding, zinc ion binding
			TraesCS2A02G153500	P-loop containing nucleoside triphosphate hydrolase, small GTP-binding protein domain	GTPase activity, GTP binding
QFeC-6B.1	6B	4.8–8.0	TraesCS6B02G012700	Guanine nucleotide binding protein (G-protein), alpha subunit, P-loop containing nucleoside triphosphate hydrolase	GTPase activity, GTP binding, G-protein beta/gamma-subunit complex binding
			TraesCS6B02G007400	Amino acid transporter, transmembrane domain	Integral component of membrane
QFeC-1D.3	1D	12.8–17.1	TraesCS1D02G036500	AP180 N-terminal homology (ANTH) domain, phosphoinositide-binding clathrin adaptor	Phospholipid binding, 1-phosphatidylinositol binding, clathrin binding
			TraesCS1D02G032500	Papain-like cysteine peptidase superfamily, cathepsin propeptide inhibitor domain	Cysteine-type peptidase activity

## Discussion

Quantitative trait loci mapping is a useful strategy to identify genomic regions governing quantitative traits and to identify molecular markers to facilitate marker-assisted breeding (MAB) of the desired trait into the elite germplasm. Identification of stable QTL across a wide range of environments is of great importance in a MAB program. The present study dissected QTL for two important nutritional traits in wheat, grain Zn and Fe concentration. We identified several stable genomic regions governing GFeC and GZnC through the application of both inclusive composite interval mapping, which identifies additive and dominant QTL in single environments, and multi-environment QTL mapping which uses QTL-by-environment interaction (QEI) model and identifies QTL with average additive effect and QEI effects (Li et al., 2015).

The parental lines in the present study showed contrasting phenotype for grain iron and zinc concentrations and agronomic traits. The high Zn parent ‘Zinc-Shakti’ is a synthetic hexaploid wheat (CROC1_/AE.SQUARROSA(210)//INQALAB 91^∗^2/KUKUNA/3/PBW343^∗^2/KUKUNA), whereas the other parent ‘Kachu’ is a high-yielding adapted line grown widely in South Asia. Both coefficient of parentage (COP = 0.17) and SNP-based diversity (π = 0.26) indicated that the parents are distantly related and this highly likely is the reason for the ample transgressive segregation observed in our population for both nutritional traits. The action of loci with complementary additive effect differentially present in parental lines can be detected when progenitors are distantly related (Rieseberg et al., 1999). In agreement with this, we detected QTL originating from both parents, signifying the complementary effect of QTL.

Kumar et al. (2007); and Wu et al. (2012) demonstrated the importance of QTL-by-environment interactions for quantitative traits and emphasized that the estimation of the main-effect QTL is biased if QEI were not examined. We detected 27 and 23 QTL in multi-environment QTL mapping and 9 and 4 QTL in ICIM analysis for GZnC and GFeC, respectively. Most significantly, all stable QTL detected in ICIM analysis were also detected in multi-environment analysis. It is, however, noteworthy that multi-environment analysis identified a lot more pleiotropic QTL for GZnC and GFeC which could not be detected in ICIM analysis. For GZnC, QTL with the highest PVE were identified on chromosomes 1B and 7D (7.7 and 8.1%, respectively), whereas for GFeC, these were detected on chromosomes 2A and 6B (10.1 and 10.2%, respectively). Previous QTL studies have also mapped QTL for GZnC and GFeC on these chromosomes (Peleg et al., 2008; Xu et al., 2012; Srinivasa et al., 2014; Krishnappa et al., 2017; Velu et al., 2017, 2018; Liu et al., 2019). Most significantly, we detected seven pleiotropic regions or overlapping regions for GZnC and GFeC in the present study on chromosomes 1B, 1D, 2B, 6A, and 7D (Figure 3). Previous studies have reported pleiotropic QTL for GZnC and GFeC on chromosomes 2B, 3B, 3D, 4B, and 5A (Xu et al., 2012; Hao et al., 2014; Crespo-Herrera et al., 2016, 2017). Hence, the identification of new pleiotropic regions for GZnC and GFeC in the current study has expanded the diversity of QTL that could be used for simultaneous improvement of GZnC and GFeC. Notably, the identification and cloning of GPC-B1 gene located on chromosome 6B an early regulator of senescence and affects remobilization of protein and minerals to the grain by Uauy et al. (2006) followed by Pearce et al. (2014) documented the GPC-B1 is a NAC transcription factor and has a paralogous copy on chromosome 2 in wheat. Apparently, some of the QTL identified in this study may have associated with the GPC-B1. Based on the best pleiotropic QTL combination, we have identified nine transgressive individuals from the RIL population that could be used in the breeding pipeline (Table 6). The higher number of pleiotropic QTL for GZnC and GFeC obtained in the present study vis-à-vis previous studies is largely due to the higher correlation obtained between GZnC and GFeC as compared with previous studies (Xu et al., 2012; Crespo-Herrera et al., 2017; Rathan et al., 2020). For example, Xu et al. (2012); and Crespo-Herrera et al. (2017) reported correlations between GZnC and GFeC ranging from 0.42 to 0.82 and 0.38–0.63, respectively, in different environments, whereas we obtained correlations ranging from 0.76 to 0.96 between the two traits in different years. Further, it is noteworthy that none of the QTL identified here had significant G × E effects as all QTL detected here had larger LOD scores for the additive average effect than the LOD score of the interaction (Table 5). The contribution of epistatic interactions in the genetic architecture of disease resistance, end-use quality, and grain yield has been extensively investigated in wheat using bi-parental designs (Zhou et al., 2002; Yang et al., 2005; Ma et al., 2006; Kumar et al., 2007; Mann et al., 2009; Singh et al., 2009; Kolmer et al., 2011; Wu et al., 2012). However, little information is available on epistatic interactions of QTL for nutritional traits (Xu et al., 2012). The current study identified significant epistatically interacting QTL for both GZnC and GFeC by two- and three-locus interactions, which suggests the significant role of epistasis in their genetic architecture. Particularly for GZnC, PVE by epistatically interacting loci was higher than PVE explained by 11 additive QTL. Hot spots of epistatic interactions were found on chromosomes 2B and 4A for GZnC and on chromosomes 2A and 7D for GFeC.

*In silico* BLAST search identified various potential candidate genes underlying QTL with high PVE or pleiotropic QTL for GZnC and GFeC (Table 7). *QZnC-2A.2* appears to overlap with a gene coding for ABC transporter. In the past years, a great wealth of information has been gained in understanding the interaction of Fe and Zn homeostasis in plants as a consequence of the chemical similarity between their divalent cations. In this regard, ABC transporters have been predicted to play an important role in plants. In *A. thaliana*, for example, the ABC transporters are shown to contribute to the accumulation of Cd–phytochelatin (Cd–PC) complexes in the vacuole. The chemical similarity of Cd^2+^ to Zn^2+^ and the important role played by phytochelatins in Zn homeostasis (Tennstedt et al., 2009) suggest that these transporters or their homologs might contribute to Zn homeostasis. The pleiotropic QTL identified on chromosome 7D displayed its location in a region where genes code for oligopeptide transporters (OPTs) family. OPTs encode integral membrane proteins that play critical roles in the transport of small peptides, secondary amino acids, glutathione conjugates, and mineral uptake (Kumar et al., 2019). The expression pattern of genes belonging to the subfamily of OPT transporters in wheat during iron starvation experiments revealed an early high transcript accumulation of a few in roots (Kumar et al., 2019). The proven role of OPTs in long-distance iron transport or signaling in *Arabidopsis* (Stacey et al., 2008) further suggests both candidates, *TraesCS7D02G099500* and *TraesCS7D02G139600*, underlying this pleiotropic QTL are strong for future validation studies. The BLAST results for *QZnC-1B.1*, another pleiotropic QTL for GZnC and GFeC, displayed its location in a region where genes code for the serine-threonine/tyrosine-protein kinase-like superfamily (Scheeff and Bourne, 2005), which catalyzes phosphorylation processes, and some are known to activate Zn channels and ZnT zinc transporters (Thingholm et al., 2020). In addition, in the region of *QFeC-1D.3*, there was a gene encoding for papain-like cysteine peptidase superfamily. Cysteine proteins act as “redox switches” and play an important role in regulatory and signaling pathways; sense concentrations of oxidative stressors and unbound zinc ions in the cytosol and control the activity of metalloproteins (Giles et al., 2003).

## Conclusion

The GZnC was found to have a highly significant positive correlation with GFeC (0.76–0.96) suggesting the possibilities of simultaneous improvement of both GZnC and GFeC concentration in wheat. Further, identification of pleiotropic regions for GZnC, GFeC, and TKW suggests the possibilities of genetic improvement of GZnC and GFeC without compromising grain yield. The novel pleiotropic QTL identified in the present study have not only expanded the diversity of QTL that could be used in wheat breeding programs but also has opened vistas of validating many underlying candidate genes for biofortification. The nine RILs identified with the best combination of pleiotropic QTL can be used in the breeding pipeline and can also serve as direct candidates of biofortified varieties.

## Data Availability Statement

The original contributions presented in the study are included in the article/Supplementary Material, further inquiries can be directed to the corresponding author/s.

## Author Contributions

NR did the QTL mapping and wrote the manuscript. DS contributed to the assistance for QTL mapping. KT contributed to the assistance on identification of candidate genes using bioinformatics. A-MS supervised the data and wrote, reviewed, and edited the manuscript. RS contributed basic germplasm and resources. VG contributed to the resources, conceptualization, developed the mapping population, and generation of genotypic data. All authors contributed to the article and approved the submitted version.

## Conflict of Interest

The authors declare that the research was conducted in the absence of any commercial or financial relationships that could be construed as a potential conflict of interest.

## Footnotes

1 http://www.isbreeding.net

2 http://wheat.pw.usda.gov/ggpages/wgc/98/Intro.htm

3 http://plants.ensembl.org/index.html

## Supplementary Material

Supplementary Figure 1 Histograms of TKW in the mapping populations of recombinant inbreed lines evaluated during 3 years, and across the years.

Supplementary Figure 2 Histograms of PH in the mapping populations of recombinant inbreed lines evaluated during 3 years, and across the years.

Supplementary Figure 3 Histograms of TW in the mapping populations of recombinant inbreed lines evaluated during 2 years, and across the years.

Supplementary Figure 4 Histograms of DH in the mapping populations of recombinant inbreed lines evaluated during 2 years, and across the years.

Supplementary Figure 5 Histograms of DM in the mapping populations of recombinant inbreed lines evaluated during 2 years, and across the years.

Supplementary Table 1 Quantitative trait loci for agronomic and yield related traits in RIL population.

Supplementary Table 2 Epistatic QTL identified by two- and three-locus interactions for grain Zn and Fe concentration.
