# Supplementary material for: Identification of Genetic Loci and Candidate Genes Related to Grain Zinc and Iron Concentration Using a Zinc-Enriched Wheat ‘Zinc-Shakti’
Source: Front Genet. 2021 May 31;12:652653. doi: 10.3389/fgene.2021.652653 (PMC8237760; doi:10.3389/fgene.2021.652653)

Supplementary figure 1: Histograms of TKW in the mapping populations of recombinant inbreed lines evaluated during 3 years, and across the years


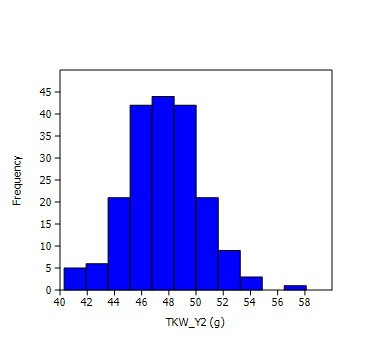

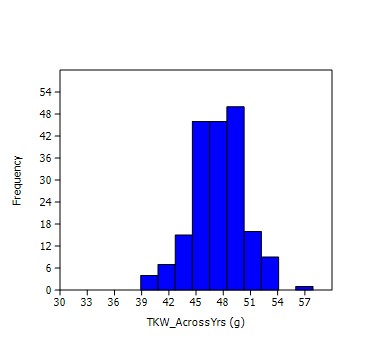

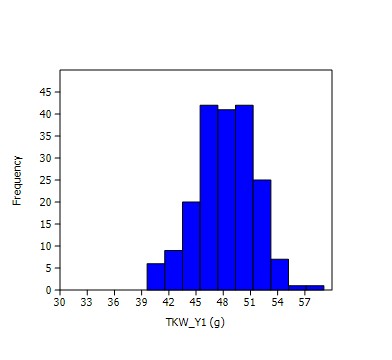

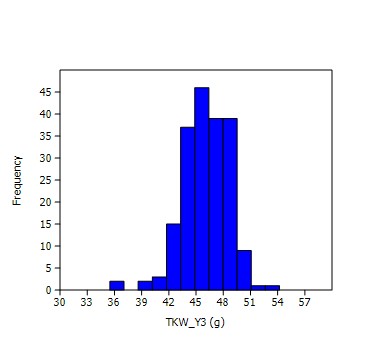


Supplementary figure 2: Histograms of PH in the mapping populations of recombinant inbreed lines evaluated during 3 years, and across the years


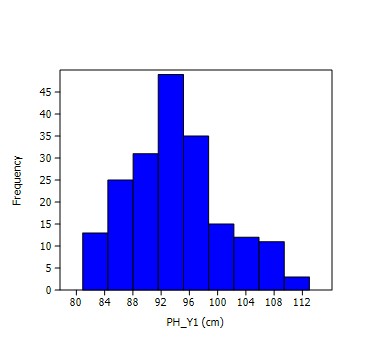

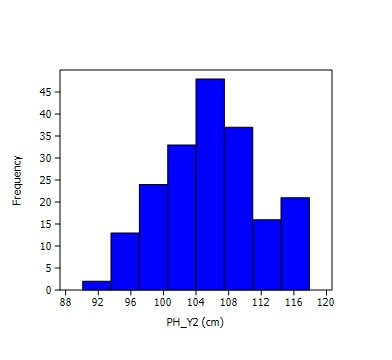

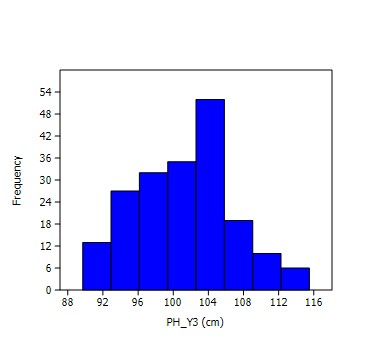

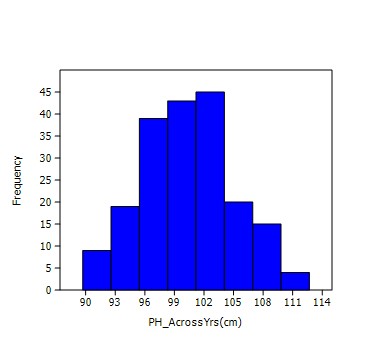


Supplementary figure 3: Histograms of TW in the mapping populations of recombinant inbreed lines evaluated during 2 years, and across the years


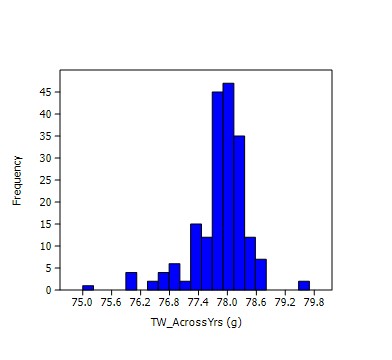

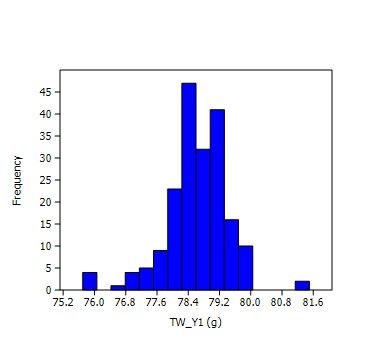

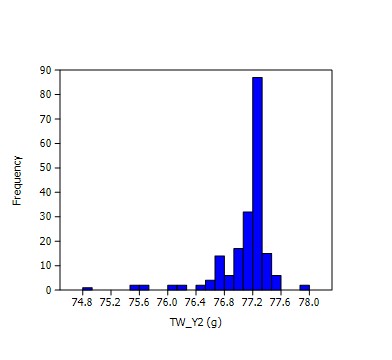


Supplementary figure 4: Histograms of DH in the mapping populations of recombinant inbreed lines evaluated during 2 years, and across the years


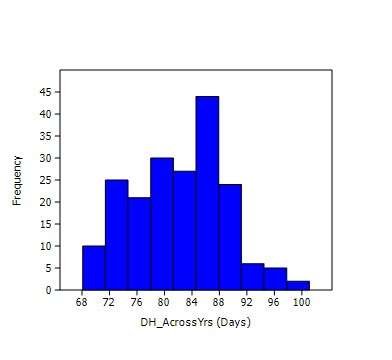

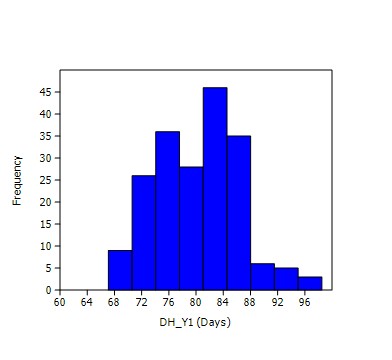

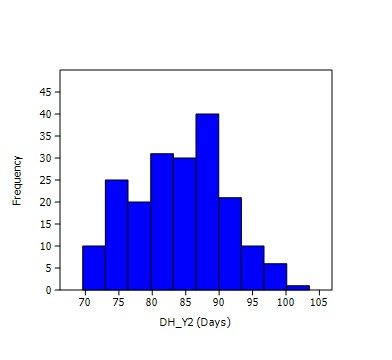


Supplementary figure 5: Histograms of DM in the mapping populations of recombinant inbreed lines evaluated during 2 years, and across the years


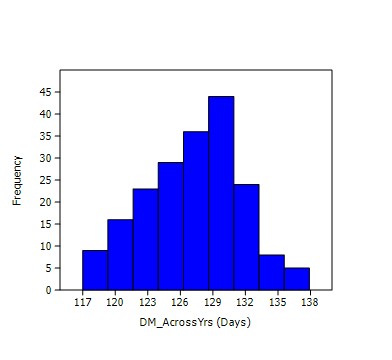

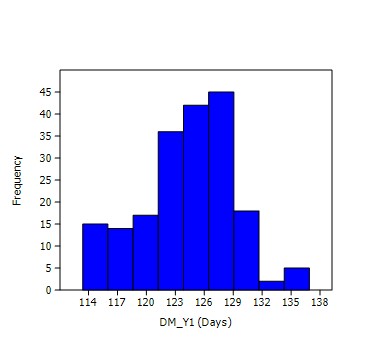

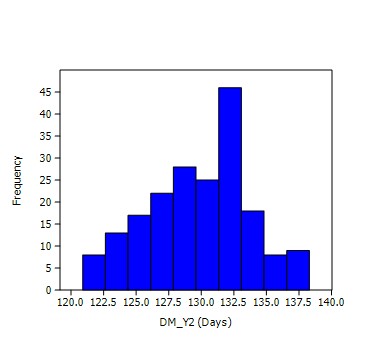

Supplement: Supplementary Figure 1 — Histograms of TKW in the mapping populations of recombinant inbreed lines evaluated during 3 years, and across the years. [file Data_Sheet_1.docx]
