# Supplementary material for: Identification of Genetic Loci and Candidate Genes Related to Grain Zinc and Iron Concentration Using a Zinc-Enriched Wheat ‘Zinc-Shakti’
Source: Front Genet. 2021 May 31;12:652653. doi: 10.3389/fgene.2021.652653 (PMC8237760; doi:10.3389/fgene.2021.652653)
Supplement: Supplementary Table 2 — Epistatic QTL identified by two- and three-locus interactions for grain Zn and Fe concentration. [file Table_2.DOCX]

Supplementary Table 2. Epistatic QTL identified by two- and three-locus interactions for grain Zn and Fe concentration.

| **Trait** | **Two locus interaction** | **PV (%) explained by two interacting loci** | **Three locus interaction** | **PV (%) explained by three interacting loci** |
| --- | --- | --- | --- | --- |
| GZnC | QZnC-2B.4  QZnC-2B.2 | 3.2 | QZnC-1D.1  QZnC-2B.2  QZnC-6A.1 | 3.2 |
|  | QZnC-2B.3  QZnC-2B.2 | 2.1 | QZnC-2B.4  QZnC-2B.2  QZnC-6A.1 | 4.6 |
|  | QZnC-2B.3  QZnC-1B.1 | 2.4 |  |  |
|  | QZnC-2B.3  QZnC-4A.1 | 2.2 |  |  |
|  | QZnC-2B.2  QZnC-6A.1 | 2.6 |  |  |
|  | QZnC-2B.2  QZnC-4A.1 | 2.4 |  |  |
| GFeC | QFeC-7D.1  QFeC-7A.2 | 1.4 | QFeC-7D.1  QFeC-1D.1  QFeC-7A.1 | 1.7 |
|  | QFeC-1D.1  QFeC-2A.1 | 1.3 | QFeC-5B  QFeC-1B.1  QFeC-6A | 1.6 |
|  | QFeC-1B.2  QFeC-2A.2 | 1.6 |  |  |
|  | QFeC-1B.2  QFeC-2A.1 | 1.9 |  |  |
